# Atopic dermatitis and food allergy: when and how to test?

**DOI:** 10.1186/2045-7022-1-S1-S25

**Published:** 2011-08-12

**Authors:** Margitta Worm

**Affiliations:** 1 Allergy-Center-Charité, Charité Campus Mitte, Universitätsmedizin Berlin, Department of Dermatology and Allergy, Berlin, Germany

## 

Atopic dermatitis is a multifactorial skin disease. Epidermal barrier dysfunction, bacterial colonisation, psychological stress, but also type I allergens may aggravate this chronic remittent skin disease. Food allergens may play a role as aggravating factors in a subgroup of patients with atopic dermatitis, however the elicitating allergen profile differs between children and adults.

In children such potential food allergens are milk, egg and wheat. These allergens are rarely of importance in adults. Here, if present, more frequently pollen associated food allergens may play a role as an aggravating factor. In principal much more patients assume allergic reactions against food being responsible for triggering eczematous reactions worsening the eczema. Therefore the identification of such patients who indeed will benefit from an elimination diet is important and will also result in the avoidance of unnecessary diets.

The gold standard for the diagnosis of food dependent reactions is the performance of a placebo controlled, double blind oral provocation test because specific IgE, skin prick test and the patients history often do not correlate with clinical reactivity. In particularly in atopic eczema such procedure is extremely important for differentiation between food as a cause of a trigger of delate eczematous skin reactions.

Before the performance of an oral provocation test a diagnostic elimination diet over a limited time frame is necessary. If several sensitisations are suspected an oligo allergenic diet and afterwards a subsequent implementation of defined food items can be performed. If the diagnostic elimination diet improves the severity of the eczema e.g. measured by SCORAD score oral provocation tests should be performed. As late phase reactions may develop slowly it is recommended to assess the SCORAD score not only the day after the provocation test but also after 24 hours followed by a second provocation if the first one did not aggravate the eczema (increase of SCORAD at least of 10 points). In particular in children the clinical relevance of food allergens should be re-evaluated every one to two years as the development of tolerance in particular towards milk is possible.

The value of the atopy patch test for the diagnosis of type I clinical relevant type I sensitisation has been excessively studied, however due to the fact that no standardised extracts for testing are available it is not recommended for daily practise.

In atopic eczema three clinical reaction patterns are possible (non-eczematous skin reactions e.g. urticaria, isolated eczematous delate skin reactions, excercabation of pre existing eczematous skin leasons after several hours or one to two days or a combination of non-eczematous early reactions and eczematous late reactions).

## Take home message

Suspected food allergy is frequent in patients suffering from atopic dermatitis, however to maintain quality of life a stepwise diagnostic approach is necessary. Double.blind placebo-controlled oral provocation is the gold standard to proof a clinical relevant food allergy in patients suffering form atopic dermatitis.
